# Five-month-old infants detect affiliation in colaughter

**DOI:** 10.1038/s41598-019-38954-4

**Published:** 2019-03-11

**Authors:** Athena Vouloumanos, Gregory A. Bryant

**Affiliations:** 1 Department of Psychology, New York University, New York, New York USA; 2 Department of Communication, University of California at Los Angeles, Los Angeles, California USA

**Keywords:** Evolution, Neuroscience

## Abstract

Colaughter–simultaneous laughter between two or more individuals–allows listeners across different cultures and languages to quickly evaluate affiliation within a social group. We examined whether infants are sensitive to acoustic information in colaughter that indicates affiliation, specifically whether they can differentiate colaughter between friends and colaughter between strangers. In the first experiment, infants who heard alternating trials of colaughter between friends and strangers listened longer to colaughter between friends. In the second experiment, we examined whether infants were sensitive to the social context that was appropriate for each type of colaughter. Infants heard colaughter between friends and colaughter between strangers preceded by a silent visual scene depicting one of two different social contexts: either two people affiliating or turning away from each other. Infants looked longer when the social scene was incongruent with the type of colaughter. By 5 months, infants preferentially listen to colaughter between friends and detect when colaughter does not match the valence of a social interaction. The ability to rapidly evaluate acoustic features in colaughter that reveal social relationships between novel individuals appears early in human infancy and might be the product of an adaptive affiliation detection system that uses vocal cues.

## Introduction

Infants begin engaging with the social environment and communicating with their caregivers within their first months of life. But infants also start observing the pattern of communicative and social behaviors between people around them^1–4^. Through infants’ third-party observations of social interactions, infants recognize affiliative intentions in social agents and use these to make predictions about others’ behavior (for review see^5^).

Infants use a variety of types of information to reason about social relationships. For example, by 9 months, infants use shared positive evaluations, such as two people liking the same food, as a cue to affiliation^6,7^. Infants at this age also infer that people who speak the same language will likely be friends^8^. Even younger infants can use an agent’s helping or hindering behavior to infer the nature of the relationship–affiliative or antagonistic–between agents^2,9^. In their second year, establishing affiliative relationships between agents through labels or actions creates expectations about how those agents will behave towards others^10–12^, allowing infants to make sense of complex social relationships.

Our understanding of infants’ inferences about others’ social behaviors comes in part from how infants perceive visual cues of affiliative interaction, such as being hindered, harmed, or helped in some attempted action. But less is known about auditory cues of affiliative interaction. Vocal communicative behavior reveals a great deal about people’s intentions, and infants are highly sensitive to intentional vocal signals such as speech e.g.,^13,14^, but little research has examined how infants might use nonverbal auditory cues to make social inferences^15,16^. Vocalizations are a particularly expressive communicative medium, including not only speech, the main channel for language use, but also emotional signaling^15^.

Laughter is a ubiquitous, nonverbal vocalization with a variety of functions, including communicating one’s intentions to positively affiliate^17,18^. Spontaneous laughter is generated by an evolutionarily conserved vocal emotion production system shared by most mammals^19,20^. Human laughter is homologous to play vocalizations in nonhuman primates, and has been suggested to be a ritualized signal of heavy breathing during rough and tumble play^17^. Chimpanzees produce laugh-like play vocalizations to prolong playing time, suggesting that chimpanzees use laughter to signal they are engaging in non-threatening playing behavior that would otherwise resemble actual fighting^21^. Laughter in humans could serve a similar function as a non-threatening signal during tickling and rough and tumble play in children^17^.

**Figure 1:**
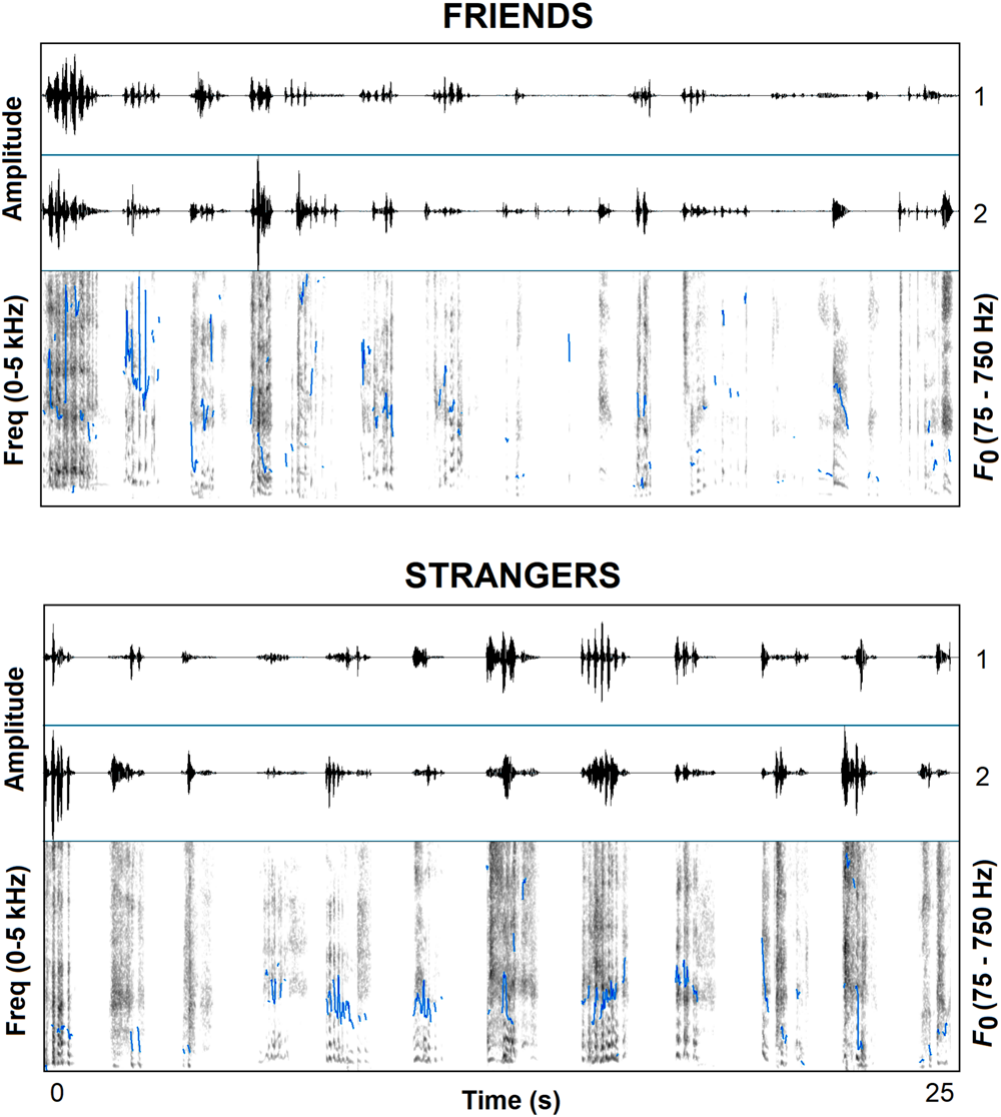
Waveform (top) and narrowband spectrogram (bottom; 30 ms Gaussian analysis window, 44.1 kHz sampling rate, 0–5 kHz frequency range) of friends ‘and strangers’ laughter. Waveform representation shows speakers 1 and 2 on their respective channels. Blue lines represent fundamental frequency (F0) values (75–750 Hz range).

But, in humans, volitional laughter has taken on a more complex and varied set of functions, such that it can manifest itself in almost any social context. In many social interactions, people often laugh together (i.e., simultaneously), and this colaughter varies with their friendship status^22^. When brief segments of colaughter between friends and strangers were presented to listeners from 24 different societies, ranging from small-scale hunter-gatherers to rural farmers to urban students, listeners everywhere were able to distinguish colaughter between friends from colaughter between strangers^23^. Acoustic analysis showed that listeners’ judgments of affiliative status were largely driven by vocal features associated with physiological arousal^23^. Specifically, laughter between friends was more likely to consist of shorter calls (i.e., faster sounding), less regular pitch and intensity cycles, and less variation in pitch cycle regularity (See Fig. 1). These acoustic differences are similar to the features that distinguish spontaneous and volitional laughter, and suggest that friends are more likely to engage in shared spontaneous laughter than strangers, something listeners can detect. Here we explore whether infants can use vocal information to infer affiliative status by distinguishing between laughter between friends and laughter between strangers. Spontaneous laughter has a long evolutionary history suggesting that humans may be particularly attuned early in development to the acoustic features associated with emotional vocal production.

Infants attend to others’ laughter, and can use adults’ laughter to understand others’ actions and intentions. Six-month-olds use laughter for social referencing, for example looking at their parents more when they used an object unconventionally (using a ball as a clown nose) while laughing, than when they used it conventionally (bouncing a ball on the ground) while laughing^24^. Infants who themselves laughed at the unconventional act looked at their parent less, suggesting they didn’t need affective support. Infants also understand some of the functional significance of laughter, linking a person’s vocal signals of humor to that person’s intentions and actions. For instance, 15-month-old infants matched a person’s humorous vocal signals (laughter and a humorous sentence) to her humorous actions^16^. Infants can thus recognize that laughter can be informative about a person’s behavior.

**Figure 2:**
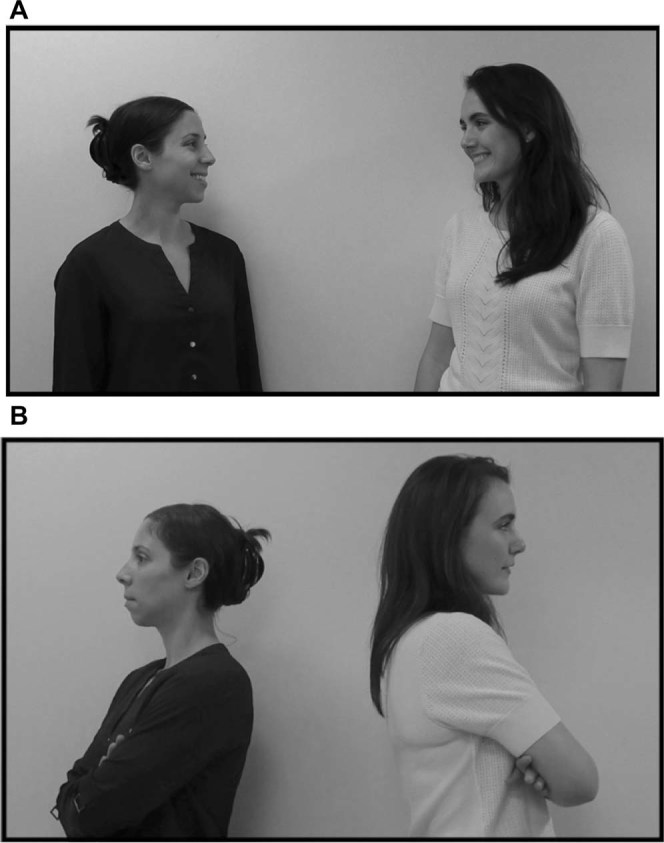
Black and white still image from the affiliative video (**A**) and the disengaged video (**B**) shown in Experiment 2.

But little work has examined how infants perceive laughter, and no work, to our knowledge, has explored whether infants can draw inferences about affiliative relationships from auditory information alone. In two experiments, we examined whether infants were sensitive to the acoustic and social signals in colaughter between friends and strangers. In the first experiment, infants heard alternating trials of laughter between friends and laughter between strangers. We expected that infants would discriminate between the two types of colaughter, and would attend preferentially to friends’ colaughter over strangers’ colaughter. In the second experiment, infants heard either colaughter between friends or colaughter between strangers, preceded by visual scenes depicting one of two different social contexts: either two actors affiliating with each other or turning away from each other (See Fig. 2). A positive affiliative condition was contrasted with a negative rather than a neutral condition to ensure that infants could differentiate and recognize the valence of the two interactions, consistent with prior studies examining infants’ perceptions of social relationships^6–8,25^. Also consistent with these prior infant studies on social relationships, we predicted that if infants recognize the social context that was appropriate to each type of laughter, they would look longer when the laughter and social context were incongruent: when stranger colaughter followed affiliative interactions, and when friend colaughter followed disengaged interactions.

## Experiment 1: Preference for Friend or Stranger Colaughter

### Method

#### Participants

Participants were 24 healthy, full term infants (12 females; *M* age: 5 months 4 days, *SD* = 12 days) recruited from maternity wards at local hospitals (New York, NY). Parents reported the ethnicity of their infants as White (13), Black (3), Asian (1), mixed race (4), or chose not to answer (3). Of these infants, 5 were identified as Hispanic or Latino, 18 were identified as non-Hispanic and 1 family chose not to answer. Parents reported their highest educational degree as some college (3), college degree (4), graduate degree (16), or chose not to answer (1). An additional 10 infants were excluded because they looked at the screen for the maximum trial length for more than 50% of the trials (5), fussiness (2), a preexisting medical condition (1), or looking times greater than 2.5 standard deviations from the mean (2). Parents gave informed consent on behalf of their infants and received a certificate and small toys or t-shirts as gifts for their participation. All procedures were approved by the IRB at New York University (IRB-FY2016-81). All experiments were performed in accordance with the relevant guidelines and regulations.

#### Stimuli

Pretest and posttest music: Before and after the experimental trials, infants saw a black and white checkerboard while hearing a clip of Bach’s Concerto for Violin and Orchestra No. 1 in A minor (BWV 1041-III. Allegro Assai). This music trial familiarized infants with the visual and sound aspects of the procedure e.g.,^26^ so that we did not have to exclude any experimental data from the analyses e.g.,^27^.

*Colaughter segments* were extracted from conversations between pairs of American English-speaking undergraduate students who participated in exchange for course credit. Participants either signed up with a friend whom they had known for any amount of time, or they signed up individually and were paired with a stranger. The participants were instructed to talk about any topic they chose. The average conversation length was 13.5 min (*M* length = 809.2 s, *SD* = 151.3). Each conversation participant wore a lapel microphone approximately 15 cm from their mouth (Sony ECM-77B) and was recorded digitally to DAT (16-bit amplitude resolution, 44.1-kHz sampling rate, uncompressed wav files, Sony DTC series recorder) on a separate audio channel. See^28^ for a complete description of the conversations.

Based on previous work^23^, we defined colaughter as concurrent laughter production (with intensity onsets within 1 s). The laughs could be either voiced (periodic) or unvoiced (aperiodic). Acoustically, laughter is variable but often characterized by an initial alerting component, stable vowel configurations, and decaying loudness and pitch^29,17,30^. From 24 conversations, two colaughter segments were extracted from each (the first and last occurrence) for a total of 48 colaughter segments. Half of the conversations were between friends (*M* length of acquaintance = 20.5 months; Range = 4–54 months; *M* age = 18.6 years; *SD* = 0.6) and half were between newly acquainted strangers (*M* age = 19.3 years, *SD* = 1.8). An analysis of basic acoustic features was performed using Praat (version 5.4.15)^31^. F0 values were calculated using an autocorrelation method with recommended pitch settings of 100–600 Hz for females and 75–500 Hz for males. Spectral measures were calculated using a cross correlation method. See Fig. 1 and Table 1 for acoustic characteristics. See^23^ for more information about the colaughter segments.

**Table 1:** Acoustic analyses of friend colaughter and stranger colaughter.

Acoustic measure	Colaughter type			
	Friends		Strangers	
Burst number	4.2	(1.8)	3.5	(1.6)
Bout length (ms)	1146	(455)	1067	(266)
Average burst duration (ms)	274	(108)	301	(99)
Laughter onset asynchrony	337	(299)	290	(209)
Mean F0 (Hz)	283	(88)	254	(79)
F0 SD (Hz)	43	(20)	32	(16)
F0 Min (Hz)	207	(67)	200	(65)
F0 Max (Hz)	377	(123)	329	(118)
F0 Range (Hz)	170	(88)	129	(77)
Center of gravity (Hz)	973	(342)	821	(427)
Loudness (dB)	59.6	(7.6)	60.3	(6.0)
Harmonics-to-noise ratio (dB)	5.5	(2.2)	6.4	(2.3)

Values reported are means with standard deviations in parentheses. Laugh bursts were counted as a combination of the two speakers. For example, simultaneous laugh bursts between two speakers counted as one burst, but if two overlapping bursts were perceptible as two bursts, they were counted as two.

Using these 48 colaughter segments (24 friend colaughter and 24 stranger colaughter), we created 4 audio files of friend colaughter and 4 audio files of stranger colaughter using Audacity 2.1.2 (Freeware distributed under GNU General Public License). Each trial included 12 laughter segments and was 24.5 s long with 300–1200 ms of silence between laughter segments.

#### Procedure

Infants were tested in a sound-attenuated room using an infant-controlled sequential preferential looking procedure (e.g.,^27,26^ run in Habit 2.1.25^32^). In this procedure, infants controlled the onset and offset of each trial by looking at or away from a central monitor. Infants sat on a caregiver’s lap 35′′ (89 cm) in front of a 30′′ (76.25 cm) computer monitor.

At the start of the experiment, infants’ attention was drawn to the monitor by a colorful expanding and contracting circle. Once infants fixated on the monitor, a stationary black and white checkerboard appeared in tandem with one set of sounds, either friend colaughter or stranger colaughter, presented at a mean amplitude of 60 dB (±5 dB). Sounds played until infants looked away from the monitor for 2 consecutive seconds, at which time the sounds and the visual display ceased.

The colorful expanding circle drew infants’ attention back to the monitor between trials. Once infants fixated on the monitor, the stationary black and white checkerboard appeared in tandem with the other set of sounds. We presented 5 trials each of friend colaughter and stranger colaughter in alternation for a total of 10 experimental trials. Half the infants heard friend colaughter first and half the infants heard stranger colaughter first.

We compared infants’ looking time to the screen during friend colaughter and stranger colaughter. Offline coders who were not aware of experimental condition did reliability coding on 20% of trials with the sound turned off using SuperCoder (Universal)^33^ and reliability was high (*r* = 0.99).

### Results

**Figure 3:**
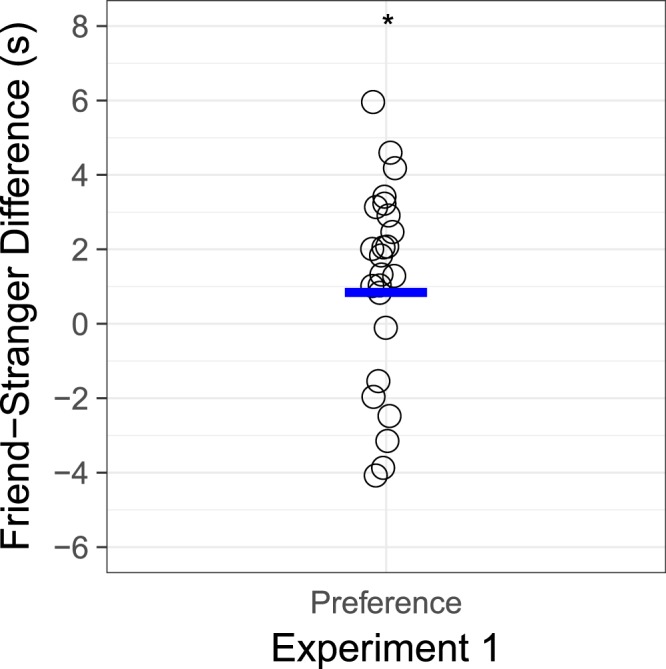
Results. Individual (circles) and mean (blue line) difference scores of listening times (in seconds) to friend colaughter and stranger colaughter in Experiment 1. * indicates a significant difference at *p* < 0.05.

Infants listened longer to friend colaughter than stranger colaughter (see Fig. 3). A laughter (2: friend, stranger) by order (2: friend first, stranger first) by trial (5) by sex (2: female, male) ANOVA with age as a covariate revealed a significant effect of laughter, with infants listening longer to colaughter between friends (*M* = 10.7 s, *SD* = 4.7) than colaughter between strangers (*M* = 9.6, *SD* = 4.1), *F*(1,19) = 4.93, *p* = 0.039, partial *η*^2^ = 0.21. A post-hoc power analysis showed that, based on this effect size of *f* = 0.52, and a sample of 24 infants, we had 66% power to detect a significant difference at an alpha level of *p* < 0.05^34^. There were no other main effects or interactions.

## Experiment 2: Detecting Social Context of Colaughter

### Method

#### Participants

Participants were 24 healthy, full term infants (8 females; *M* age: 5 months 11 days, *SD* = 11 days) recruited from maternity wards at local hospitals (New York, NY). Parents reported the ethnicity of their infants as White (15), mixed race (6), or chose not to answer (3). Of these infants, 2 were identified as Hispanic or Latino, 14 identified as non-Hispanic and 8 families chose not to answer. Parents reported their highest educational degree as finished high school (1), college degree (8), graduate degree (11) or chose not to answer (4). An additional 19 infants were excluded because they looked at the screen for the maximum trial length for more than 50% of the trials (11), a preexisting medical condition (1), technical problems (2), experimenter error (4), or looking times greater than 2.5 standard deviations from the mean (1). This higher than expected attrition rate was driven by the very large number of infants (11) who did not disengage from the display on more than half of the trials and therefore could not give a reliable differential looking time measure. Parents gave informed consent on behalf of their infants and received a certificate and small toys or t-shirts as gifts for their participation. All procedures were approved by the IRB at New York University (IRB-FY2016-81).

#### Stimuli

Visual displays consisted of 2-part videos with 2 female actors: In the first part, the actors engaged in a silent 6-s interaction in which they either acted affiliatively or disengaged towards each other (See Fig. 2). In the second part, which was presented immediately after the first part, the actors faced forward in a still frame (identical in both conditions) while co-laughter played. In the affiliative video sequence, the actors began side-by-side facing forward, turned toward each other, smiled and waved, and the scene faded to black. In the second part, the actors were shown in a still frame, facing forward toward the infant while either the congruent friend colaughter or the incongruent stranger colaughter played. In the disengaged video sequence, the actors began side-by-side facing forward, turned toward each other, immediately turned their back on each other and crossed their arms, and the scene faded to black. In the second part, the actors were shown in a still frame, facing forward toward the infant while either the congruent stranger colaughter or the incongruent friend colaughter played. The same auditory stimuli as in experiment 1 were used.

#### Procedure

Each trial had two parts:(1) a 6-s fixed length segment during which infants saw either the affiliative interaction or the disengaged interaction between the two actors, (2) a 24-s infant-controlled segment during which infants saw a still frame of the two actors facing forward while one of the laughter files was played. Infants either saw the affliliative or the disengaged interaction for each of the 10 experimental trials but the type of laughter alternated such that each infant heard 5 trials of friend colaughter and 5 trials of stranger colaughter. We compared infants’ looking time to the screen during socially congruent trials (affiliative interaction followed by friend colaughter, disengaged interaction followed by stranger laughter) with socially incongruent trials (affiliative interaction followed by stranger colaughter, disengaged interaction followed by friend laughter). Offline coders who were not aware of experimental condition did reliability coding on 20% of trials with the sound turned off using SuperCoder (Universal)^33^ and reliability was high (*r* = 0.93).

### Results

**Figure 4:**
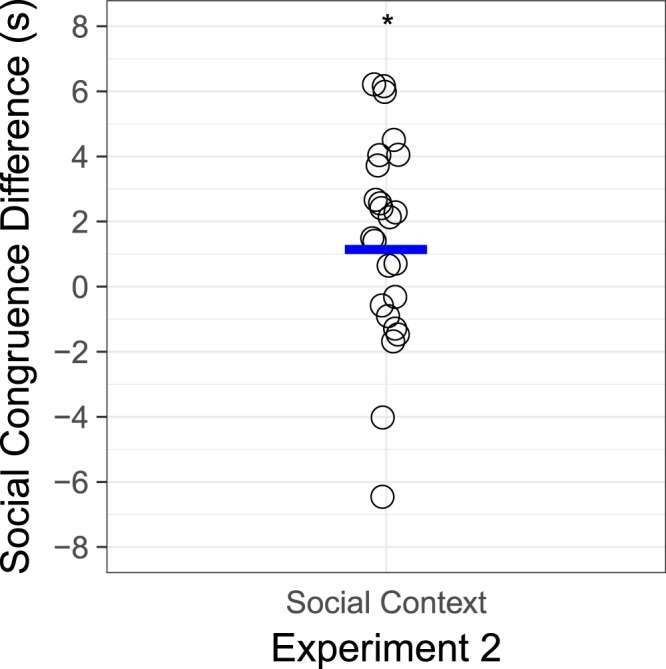
Results. Individual (circles) and mean (blue line) difference scores of listening times (in seconds) to congruent and incongruent vocal and social cues in colaughter in Experiment 2. * indicates a significant difference at *p* < 0.05.

Infants listened longer to incongruent trials than congruent trials, suggesting they noticed the discrepancy between cues in the social interaction and cues in the laughter vocalizations (see Fig. 4). A congruency (2: match, mismatch) by scene (2: affiliative, disengaged) by order (4: friend first, stranger first) by trial (5) by sex (2: female, male) ANOVA with age as a covariate revealed a significant effect of congruency, with infants listening longer to incongruent laughter (*M* = 14.0 s, *SD* = 4.0) than congruent laughter (*M* = 12.6, *SD* = 4.8), *F*(1,19) = 4.92, *p* = 0.039, partial *η*^2^ = 0.21, and a significant effect of trial, with infants listening less in later trials, *F*(4,16) = 5.28, *p* = 0.001, partial *η*^2^ = 0.22. There were no other main effects or interactions. A post-hoc power analysis showed that, as in Experiment 1, based on this effect size of *f* = 0.52, and a sample of 24 infants, we had 66% power to detect a significant difference at an alpha level of *p* < 0.05^34^. There were no other main effects or interactions.

## General Discussion

Vocal signals can quickly allow listeners to infer affiliative status between multiple individuals. Laughter constitutes an evolutionarily ancient, cross-culturally recognized social signal of affiliative status between people e.g.,^23^. We found that 5-month-old infants prefer to listen to colaughter between friends over colaughter between strangers. A different group of infants recognized the social context that was appropriate to each type of laughter, and looked longer when the type of laughter and social context were incongruent. These results provide the first evidence that infants can use a nonverbal vocal signal of affiliation in making social judgments as a third-party observer.

Infants’ sensitivity to the social information in laughter by 5 months suggests early emerging, and possibly evolutionarily conserved, perceptual machinery for evaluating potential social signals that may be used to quickly draw inferences about the relationships between agents in the social environment. These findings are consistent with a growing body of work showing that infants attend to a range of information in establishing the social relationships between agents e.g.,^11,12,6,8,2,35^.

Laughter is rich with acoustic features that infants could have used as the basis for their preference in Experiment 1. Both sets of colaughter were used in a previous study^23^, and an extensive acoustic analysis revealed that colaughter judged as being between friends had more characteristics associated with speaker arousal, including frequency and temporal irregularities, and faster call rate. The descriptive acoustic analysis performed for the current study revealed that friends’ colaughter was higher in mean *F*_0_ and *F*_0_ variability, with also slightly higher maximum *F*_0_ values (see Table 1). These *F*_0_ properties (perceived as pitch) are also shared by infant-directed speech that adults often use to address infants, and that expresses positive emotion to infants and adults^27,36,37^. Friend colaughter may have thus sounded more positive in affect than stranger colaughter to infants, and could provide a proximate explanation for why infants preferred friend colaughter in Experiment 1. During the test trials of Experiment 2, infants heard laughter while seeing a still image of the actors, which suggests that those actors were not currently producing the laughter infants heard. This may suggest a more basic association between the visual scene and the laughter, rather than expecting the actors to laugh in a particular way. Moreover, infants might be sensitive to the positive affect common to both the affiliative scene and the colaughter between friends, providing them with a basis for matching the social information in the two modalities in Experiment 2. This kind of perceptual sensitivity allows infants to begin to make predictions about important social information about others, such as who cooperates, and who is allied.

Whereas acoustic features in laughter can be informative about social affiliation, not all vocalizations appear to be informative about others’ social relationships. For example, hearing others’ conversation does not allow adults to predict their future actions in a behavioral economic game^38^. Conversely, colaughter potentially signals rich information about people’s relationships, and is associated with actual cooperative behavior, at least in males^39^, as well as being associated with other indicators of social engagement^38,40^. Infants are sensitive to voices^41,42^, and to particular tones of voices that signal positive affect and intentions to communicate (e.g., infant-directed speech and song;^43–45^) and, like adults may be able to use acoustic features in speech to infer social relationships.

The current work reveals that infants can extract meaningful information from laughter about others’ social relationships. Our findings suggest that infants can distinguish specific acoustic properties of laughter that index others’ social relationships (as friends or strangers) and can match colaughter to the affiliative status (affiliative or disengaged) of the interlocutors. Infants’ sensitivity to different kinds of laughter might be one of the early emerging tools they use to understand and navigate the complex social world.

## Data Availability

Data are publicly available on the Open Science Framework https://osf.io/b43c8.
