# Publisher Correction: Five-month-old infants detect affiliation in colaughter

**DOI:** 10.1038/s41598-019-47826-w

**Published:** 2019-08-16

**Authors:** Athena Vouloumanos, Gregory A. Bryant

**Affiliations:** 1 Department of Psychology, New York University, New York, New York USA; 2 Department of Communication, University of California at Los Angeles, Los Angeles, California USA

Correction to: *Scientific Reports* 10.1038/s41598-019-38954-4, published online 11 March 2019

Due to a production error, the version of Figure 1 originally published with this Article contained irrelevant text. This has now been corrected in the PDF and HTML versions of the paper.

